# Effect of statins on cardiovascular complications in chronic kidney disease patients: A network meta-analysis

**DOI:** 10.1097/MD.0000000000020061

**Published:** 2020-05-29

**Authors:** Seun Deuk Hwang, Kipyo Kim, Yoon Ji Kim, Seoung Woo Lee, Jin Ho Lee, Joon Ho Song

**Affiliations:** a Division of Nephrology and Hypertension, Department of Internal Medicine, Inha University School of Medicine, Incheon; b Division of Endocrinology and Metabolism, Department of Internal Medicine, Mediplex Sejong Hospital, Incheon; c Division of Nephrology, Department of Internal Medicine, Leesin Hemodialysis and Intervention Clinic, Busan, South Korea.

**Keywords:** all-cause mortality, cardiac event, chronic kidney disease, network meta-analysis, statins

## Abstract

**Background:**

The rates of cardiovascular mortality and morbidity are increased in advanced chronic kidney disease **(**CKD). Mild to moderate CKD is associated with an increase in cardiovascular events. This study aims to investigate the effects of statins on patient mortality and cardiac events.

**Study appraisal and synthesis methods:**

Studies on statins (atorvastatin, rosuvastatin, fluvastatin, lovastatin, pravastatin, simvastatin, and simvastatin + ezetimibe) in Cochrane Central Register of Controlled Trials (CENTRAL), MEDLINE, EMBASE, and Science Citation Index Expanded databases from 1970 to February 2019 were analyzed. Inclusion criteria were randomized control trials and adult patients (>18 years old). Reviews, observational studies, and clinical trials that did not clearly define outcomes or that did not have thrombosis as an outcome were excluded. We performed direct and indirect network meta-analysis using Bayesian models and ranked different statins using generation mixed treatment comparison (GeMTC) and Stata version 13. The Grading of Recommendations Assessment, Development, and Evaluation (GRADE) of network meta-analysis approach specified 4 levels of certainty for a given result: high, moderate, low, and very low. The outcomes were cardiac events, cardiac mortality, and all-cause mortality.

**Results:**

Nineteen studies (45,863 patients) were included. Compared with placebos, pravastatin 40 mg group showed a significantly lower patient mortality (odds ratio 0.66 [95% credible interval, 0.46–0.91]).

Atorvastatin 80 mg, fluvastatin 40 mg, lovastatin 20 mg, pravastatin 40 mg, and simvastatin 40 mg showed significant results in reducing cardiac events.

In rank probability, pravastatin showed the best effect at all-cause mortality rate. Lovastatin, fluvastatin, and pravastatin showed good effects in the 1st, 2nd, and 3rd ranks in cardiac events.

**Conclusions and implications of key findings:**

Pravastatin 40 mg demonstrated the best effect on all-cause mortality, and was observed to be effective with high ranking in cardiac events. We anticipate that the data of this study will assist physicians in making informed decisions when selecting statins, such as pravastatin, as a treatment option for CKD patients.

## Abstract 2

Supplemental Digital Content is available in the text

## 1 Introduction

Patients with chronic kidney disease (CKD) have a high prevalence of cardiovascular disease (CVD), leading to increased mortality and morbidity.^[1]^ Patients with CKD show elevated levels of triglycerides in the blood, decreased levels of high density lipoprotein cholesterol (HDL-C), and changes in low-density lipoprotein cholesterol (LDL-C).^[2]^ Dyslipidemia is considered a major cause of CVD in patients with CKD; thus, its management plays an important role in the treatment of these patients.^[3]^ Although atherosclerosis is a major cause of CVD in the general population, vascular calcification due to hyperphosphatemia and secondary hyperparathyroidism in patients with CKD further increase the risk of CVD. Vascular calcification reduces blood vessel elasticity, induces hypertension, and has antagonistic effects on stenosis and thrombogenesis.^[4]^ CKD is a risk factor for CVD. Moreover, smoking and conditions such as diabetes, hypertension, hyperlipidemia, and history of thrombosis are also classified as risk factors for CVDs. Increased total cholesterol, particularly LDL-C, is a risk factor for atherosclerosis, thus, increasing the incidence of CVD.^[5]^ For treatment and management of these diseases, hydroxymethylglutaryl coenzyme A (HMG CoA) reductase inhibitors or statins are recommended as primary treatment agents besides lifestyle changes such as dietary control and appropriate exercise. The mechanism of action of statins involves blockage of the HMG CoA conversion to mevalonate during cholesterol synthesis in hepatocytes and an increase in the number of LDL receptors on the cell surface to reduce serum LDL-C.^[6]^ Compared with other drugs, statins are known to have better lipid-lowering effects. According to the National Kidney Foundation Kidney Disease Outcomes Quality Initiative (NKF-K/DOQI) guidelines, the LDL-C level for patients with diabetes mellitus and CKD should be less than 100 mg/dL.^[7]^ The guidelines recommend statin therapy for LDL-C levels >100 mg/dL. Although studies on individual statins have been published and several statins have been used in clinical practice, comparative studies on these drugs have been lacking. Herein, we performed a network meta-analysis to compare the CVD reduction effects of different statins administered to patients with CKD. We anticipate that the findings of this study will assist practitioners in selecting statins for patients with CKD.

## 2 Methods

### 2.1 Ethics statement

All results are shown in accordance with the guidelines of Preferred Reporting Items for Systematic Reviews and Network Meta-Analyses (PRISMA) statement (S1 Checklist).^[8]^ All analyses were based on previously published studies; therefore, ethical approval and patient consent were not required.

### 2.2 Data sources, searches, and inclusion and exclusion criteria

**Figure 1:**
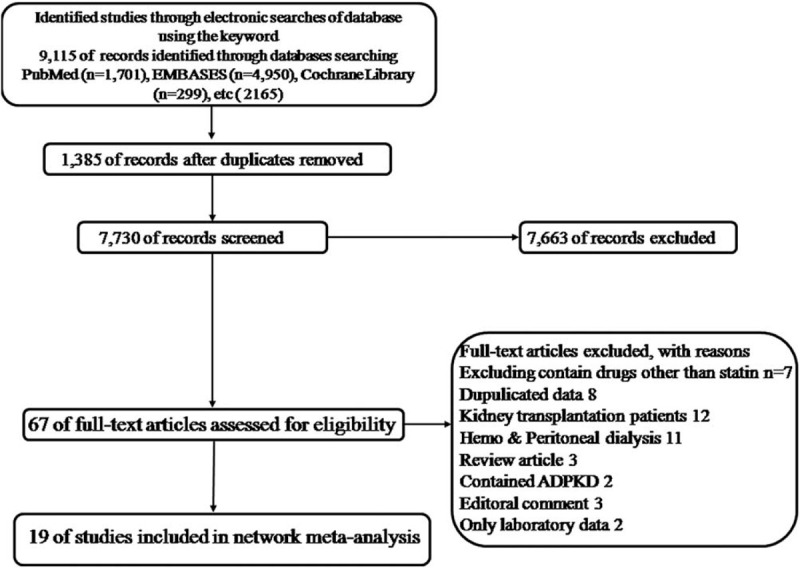
Flow diagram of the current systematic review (PRISMA flow diagram).

We performed comprehensive searches of the following databases from inception of the database until February 21, 2019: MEDLINE (via PubMed), EMBASE, CINAHL, Web of Science, and the Cochrane Central Register of Controlled Trials (CENTRAL) in the Cochrane Library. Using a highly sensitive search strategy to identify randomized control trials (RCTs), we searched for important keywords according to patient group and intervention. (Supplement 3, http://links.lww.com/MD/E143.) The criteria for including studies in the review were as follows: RCTs and adult patients (>18 years old). Reviews, observational studies, and clinical trials that did not clearly define outcomes or that did not have thrombosis as an outcome were excluded.^[9]^ The search was limited to human studies but was not restricted to any particular language or publication date. Reference lists from all available review articles and RCTs were searched manually (Fig. 1).

### 2.3 Study selection and data extraction

**Figure 2:**
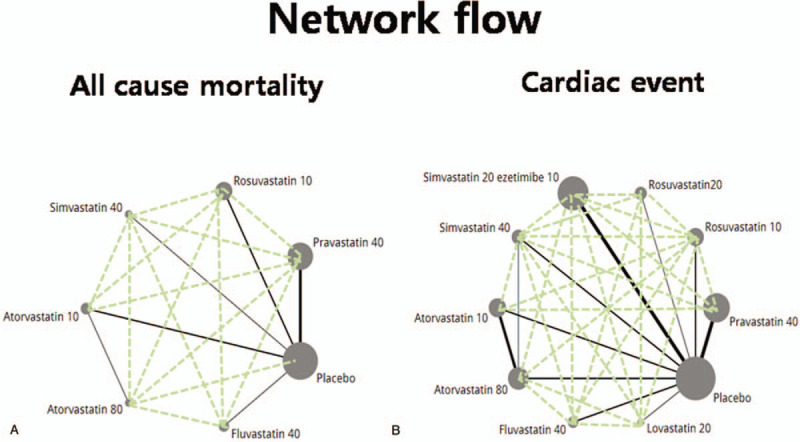
Network flow among each intervention as all-cause mortality (A) and cardiac events (B).

The abstracts and full texts identified were independently evaluated by 2 researchers (SDH and JHL). Two reviewers extracted and re-evaluated data extraction. Any disagreements were resolved through discussions and consultations with another researcher, (JHS). Inclusion criteria for the papers used in the analysis were as follows:

(1) randomized controlled studies;
(2) studies referring to at least 2 of the following eligible statins: placebo, lovastatin, atorvastatin, rosuvastatin, pitavastatin, pravastatin, simvastatin + ezetimibe, simvastatin, atorvastatin, and fluvastatin; and
(3) studies that reported one or more of the primary or secondary outcomes (Fig. 2).

### 2.4 Risk of bias assessment

Two researchers (SDH and JHL) independently assessed the risk of bias of each trial using the Cochrane Collaboration's Risk of Bias tool.^[10]^ We assessed the risk of bias during random sequence generation, allocation concealment, blinding of participants and personnel, blinding of outcome assessment, analysis of incomplete outcome data, selective reporting, and in other areas. All these judgments were categorized as “yes” (low risk of bias) or “unclear” or “no” (high risk of bias).^[10,11]^

### 2.5 Quality of evidence assessment

**Figure 3:**
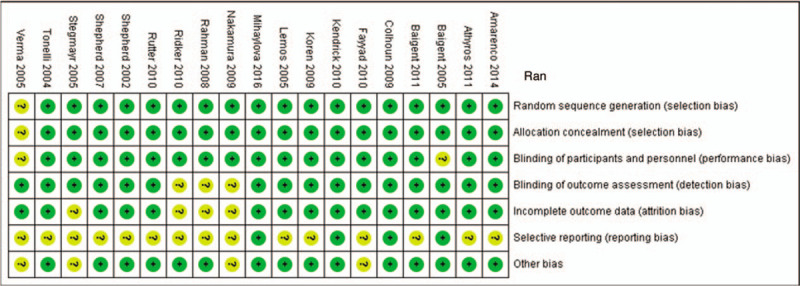
Risk of bias graph and summary of our assessment of each risk of bias item for each included study. (+) green circle, good; yellow circle, moderate; (−) red circle, bad.

We assessed the overall quality of the evidence for our primary outcomes using an adapted Grading of Recommendations Assessment, Development, and Evaluation (GRADE) approach.^[12]^ The quality of the evidence for a specific outcome was based on the performance versus the limitations of the study design, inconsistency of results, indirectness of evidence, imprecision of results, and publication bias among all studies measuring a particular outcome. The overall quality of the evidence for the outcome was produced by combining assessments from all domains (Fig. 3).^[13]^

### 2.6 Outcome measures

We aimed to determine the effectiveness of eligible statins against patient mortality and the occurrence of cardiac events. We also investigated the potential of adverse outcomes associated with these medications and assessed the efficacy of the medications on any heart failure, stroke, hospitalization, peripheral artery disease, change in LDL-C and renal function, and adverse events such as elevations in alanine aminotransferase, aspartate aminotransferase, hematuria, albuminuria, or myopathy.

Cardiac events considered in this study included death from coronary heart disease, non-fatal acute myocardial infarction, resuscitation after cardiac arrest, cardiac revascularization, and hospital admission for unstable angina.^[14]^

### 2.7 Statistical analyses

We compared statin effectiveness in terms of patient survival, cardiac event, and adverse outcomes among 10 types of statin therapy for CKD by Bayesian network meta-analysis. We performed direct and indirect network meta-analysis using Bayesian models and generated rankings of different statin agents using generation mixed treatment comparison (GeMTC) and Stata version 13 (StataCorp).^[15–17]^ The relative ranking probability of each treatment was estimated, and the treatment hierarchy of competing interventions was obtained using rankograms, surface under the cumulative ranking (SUCRA) curves, and mean ranks. We performed network meta-analysis on studies that were recorded on multiple treatments, which allows estimation of the pooled effects within each treatment.^[18]^ For multi-arm trials, correlations among the treatment effects between arms were included in the investigations. Studies with *j* + 1 treatment arms are based on comparison of the treatment effects with the reference treatment through multivariate normal distribution, whereas the treatment as usual (TAU) studies are based on the homogeneity between study variances across treatments.^[19,20]^ Inconsistency tests, homogeneity analysis, and sensitivity analysis were performed using the node analysis method in R software. The results of inconsistency tests were assessed according to the Bayesian *P*-value, where *P* < .5 was considered evidence for the existence of significant inconsistency.^[21,22]^ An *I*^2^ test was performed (*I*^2^ > 50% is considered as the existence of significant heterogeneity) to assess homogeneity. Furthermore, sensitivity analysis was conducted by comparing the differences found between fixed-effect and random-effect models. The clinical outcome indicators were evaluated using the mean difference (MD) or the odds ratio (OR) with a 95% confidence interval (CI) (MD for continuous outcomes, OR for binary outcomes).^[19,23]^ When a loop connected 3 treatments, it was possible to evaluate the inconsistency between direct and indirect evidence.^[24]^ We also used the node-splitting method to calculate the inconsistency of the model, which separated evidence for a particular comparison into direct and indirect evidence.^[22]^ We then evaluated the agreement between the direct and indirect evidence and reported its Bayesian *P* value. Sensitivity analyses were carried out using the same methods, after omission of data obtained from specific studies (studies with a small number of patients and events in a specific treatment arm, and studies with a large population that may dominate the data of specific treatment arms).^[25]^

## 3 Results

A total of 9115 records were initially retrieved from the electronic database search, from which we removed 1385 duplicate records. Of the remaining records, 7663 were excluded based on a review of either the title or abstract and 67 records were retrieved for full-text review. Among these, 46 were excluded based on the following criteria: studies involving drugs other than statins (n = 7), duplicated data (n = 8), patients who have undergone kidney transplantation (n = 12), and hemodialysis and peritoneal dialysis (n = 11), ADPKD (n = 2), an outcome that could not be included in the statistics (n = 2); review articles (n = 3); or editorial comments (n = 3).

Finally, 19 trials reporting outcomes for 45,863 patients (24,373 women and 21,490 men) were included in the analysis (Table 1). Six studies each were conducted in United States^[26–31]^ and the United Kingdom,^[32–37]^ whereas 1 study was conducted in each of the following countries: Norway,^[38]^ Japan,^[39]^ Canada,^[40]^ Scotland,^[41]^ the Netherlands,^[42]^ Sweden,^[43]^ and Greece.^[44]^ The number of patients per study ranged from 91 to 9180, and the median follow-up period was 2.1 years (0.5–3.1 years).

**Table 1:**
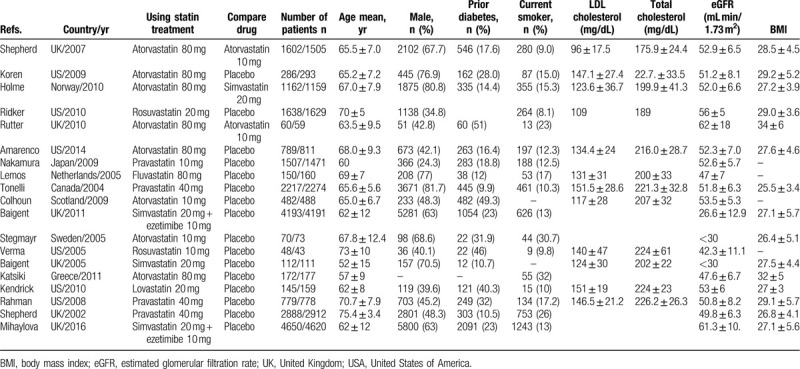
Important characteristics of the included studies and proportions of patients with using statin treatment.

### 3.1 Risk of bias in included studies

Although all the included studies were described as randomized, few gave specific details of either the method of randomization or concealment of allocation. For all studies, the blinding of study subjects and investigators was considered adequate.

Risks of bias were frequently low (Figs. 3 and S1). There were 18 (94%) studies that reported low-risk methods for random sequence generation and adequately concealed allocation; while 16 (84%) studies showed low-risk for blinding of outcome assessment (detection bias). However, only 3 studies (15%) reported low risk in selective reporting among studies. The grades of all-cause mortality and cardiac events started with high quality and well-made RCT, which then decreased by 1 point owing to the possibility of reporting bias. Thus, scientific evidence showed moderate quality results. The findings were added to the results in the manuscript.

### 3.2 Effect of interventions

**Figure 4:**
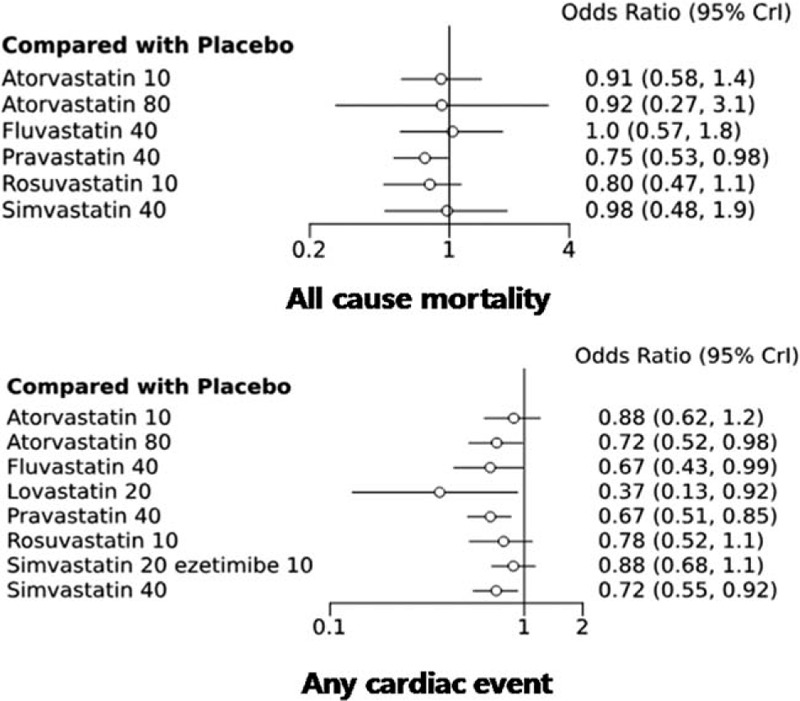
All-cause mortality (A) and cardiac events (B) associated with different types of statins and doses compared with placebos used as references.

The data obtained from all 19 studies (n = 45,863) contributed to the network analysis. The primary endpoint was patient survival. Compared with a placebo as the reference, pravastatin 40 mg significantly reduced patient mortality (OR 0.75, 95% CrL 0.53–0.98) (Table 2). However, atorvastatin 10 mg (0.91, 0.58–1.40), atorvastatin 80 mg (0.92, 0.27–3.1), fluvastatin 40 mg (1.00, 0.57–1.80), rosuvastatin 10 mg (0.80, 0.47–1.10), and simvastatin 40 mg (0.98, 0.48–1.90) were not associated with higher benefits than the placebo. No differences were found among the different interventions on the results for secondary outcome, which included myocardial infarction, heart failure, stroke, hospitalization, peripheral artery disease, a change in LDL-C and renal function, and adverse events. A placebo was used as the reference point in cases of any cardiac event. Atorvastatin 80 mg (OR 0.72, 95% CrL 0.52–0.98); fluvastatin 40 mg (0.67, 0.43–0.99); lovastatin 20 mg (0.37, 0.13–0.92); pravastatin 40 mg (0.67, 0.51–0.85); and simvastatin 40 mg (0.72, 0.55–0.92) were associated with lower ORs than the placebo in reducing cardiac events. Atorvastatin 10 mg (0.88, 0.62–1.2); rosuvastatin 10 mg (0.78, 0.52–1.1); and simvastatin 20 mg + ezetimibe 10 mg (0.88, 0.68–1.10) showed no significant difference (Fig. 4).

**Table 2:**
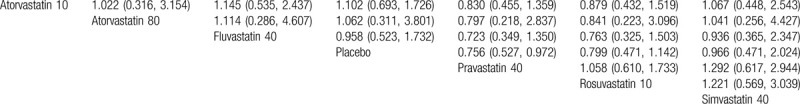
Comparison of the included statins for all-cause mortality: odds ratio (95% CI). Each cell indicates the effect of the column-defining intervention relative to the row-defining intervention.

The side effects observed subsequent to statin therapy showed no differences among the medications in terms of persistent elevations in alanine aminotransferase and/or aspartate aminotransferase, persistent elevation in creatine phosphokinase, hematuria, albuminuria, or myopathy.

### 3.3 Rank probabilities

Pravastatin 40 mg ranked first and second with probabilities of 0.292 and 0.345, respectively, for all-cause mortality; the model-fit statistic of DIC was 43.0, and the residual deviance was 22.8. The rank probabilities of cardiac events showed that lovastatin 20 mg had the highest ranking (0.824). Second in the rank probabilities was fluvastatin 40 mg with a probability of 0.309, whereas pravastatin 40 mg ranked third with a probability of 0.265. Model-fit statistic of DIC of any cardiac event was 84.8, and the residual deviance was 45.3. The network heterogeneity was estimated by comparing a common heterogeneity variance (tau [*τ*]) within each network with an empirical distribution of heterogeneity variances. The network for all-cause mortality indicated the presence of substantial heterogeneity with *τ* = 0.86, whereas *τ* = 0.74 for networks for cardiac event.

## 4 Discussion

In this study, statistically significant reduction in mortality was only observed in the groups receiving pravastatin 40 mg. Lovastatin 20 mg, fluvastatin 40 mg, and pravastatin 40 mg were ranked first, second, and third in terms of cardiac events, respectively.

This study evaluated and confirmed the potency and effectiveness of individual statins, through network meta-analysis, to reduce patient mortality and cardiac events in patients with CKD. Previous studies, however, focused only on a few representative statins, while this study identified the benefits and effects of almost all commercially available statins and their ability to reduce LDL. In these studies, statistically significant reduction in mortality was only observed in the groups receiving pravastatin 40 mg. Lovastatin 20 mg, fluvastatin 40 mg, and pravastatin 40 mg, were ranked first, second, and third, in terms of cardiac events, respectively.

Lovastatin is metabolized in the CYP3A4 pathway and has a short half-life. It was found to be advantageous in primary prevention in Air Force/Texas Coronary Atherosclerosis Prevention Study (AFCAPS/TexCAPS) clinical trials.^[7,45]^ Fluvastatin also has a short half-life. It is metabolized by CYP2C9, and has a low drug-interaction potential, except with fluconazole (CYP2C9 inhibitor) and warfarin. In the ALER trial, fluvastatin reduced the incidence of cardiovascular events in patients after kidney transplantation; none of the patients developed rhabdomyolysis.^[46,47]^ Pravastatin, a hydrophilic statin, has a short half-life, which is unaffected by food intake. It has shown cardiovascular benefits in patients with coronary artery disease in the Cholesterol and Recurrent Events (CARE) and Long-term Intervention with Pravastatin in Ischemic Disease (LIPID) trial, high-risk primary prevention West of Scotland Coronary Prevention Study (WOSCOPS), and Prospective Study of Pravastatin in the Elderly at Risk (PROSPER) trial.^[36,48–50]^ Simvastatin has a short half-life and is metabolized by CYP3A4 and can be administered without food. It was reported to reduce total mortality in both the Scandinavian Simvastatin Survival Study (4S) and HPS (Heart Protection Study).^[51,52]^ Simvastatin and ezetimibe are often administered in combination; when co-administered with simvastatin, ezetimibe prevents intestinal absorption of cholesterol and reduces LDL by 15%. Atorvastatin has a long half-life and is metabolized by CYP3A4. It has been shown to be both safe and effective in many trials, such as the TNT (Treating to New Targets), Incremental Decrease in End Points through Aggressive Lipid Lowering (IDEAL), Angle-Scandinavian Cardiac Outcomes Trial (ASCOT), and Collaborative Atorvastatin Diabetes Study (CARDS).^[53–56]^ Rosuvastatin, a hydrophilic statin with similar structure to other synthetic statins, has a long half-life (20–24 h). It has no significant CYP drug interactions and is metabolized to a minimum. In the JUPITER clinical trial, rosuvastatin significantly reduced the frequency of cardiovascular events compared with the placebo group.^[57]^ Pitavastatin, a newly synthesized statin, is almost completely metabolized by CYP2C9, resulting in very low drug interactions. It showed a good lipid-lowering effect in the Randomized Evaluation of Aggressive or Moderate Lipid Lowering Therapy with Pitavastatin in Coronary Artery Disease (REAL-CAD) study.^[58]^

Among statins, pravastatin, lovastatin, fluvastatin, and simvastatin have shorter half-lives than rosuvastatin and atorvastatin. The half-lives of rosuvastatin and atorvastatin range from 10 to 20 h, whereas the half-lives of statins that reduced cardiovascular events were 1 to 3 h. We hypothesized that these outcomes could occur in patients with CKD (and not in general population), because they have delayed metabolism and excretion, which may interfere with other medications, particularly antiplatelet agents, thus, reducing the drug effects. Most of the statins are metabolized via CYP3A4 and CYP2C9 in the liver, except pravastatin. This could be beneficial in reducing all-cause mortality and cardiovascular events because pravastatin has low level of interaction with other medications administered simultaneously, even at high doses.

A meta-analysis of randomized clinical trials in a healthy population (without CKD) revealed that statin therapy significantly reduced the risk of death, ischemic stroke, and coronary artery revascularization due to myocardial infarction or coronary heart disease.^[46,59]^ When CKD progresses to stage 4, other cardiovascular pathologies (including vascular stiffness and calcification, structural heart disease, and sympathetic hyperplasia) may develop, which contribute to an increased risk of cardiac arrhythmia and heart failure.^[48]^ Patients with CKD have a significantly higher prevalence of CVD than the general population.^[1]^ Their plasma lipid profile is characterized by increased levels of triglycerides, decreased levels of HDL-C, and inconsistent change in LDL-C.^[3]^ Statins may alleviate cardiovascular diseases through their lipid-lowering potential or via their pleiotropic effects, which alter expression of endothelial nitric oxide synthase, stability of atherosclerotic plaques, production of inflammatory cytokines and reactive oxygen species, reactivity of platelets, and development of cardiac hypertrophy and fibrosis.^[60,61]^ Recently, it was reported that statins reduced the incidence of CVD by reducing oxidant stress and inflammation at an early stage of CKD.^[62]^ Moreover, animal studies have shown that statins reduce the incidence of CVD indirectly by reducing the rate of CKD progression. Two meta-analysis studies showed that statins lower the rate of estimated glomerular filtration rate (eGFR) decline.^[63,64]^ However, in a recent systematic review, statins were shown to have no significant effect on the reduction of GFR in patients with diabetes, hypertension, or glomerulonephritis; this was only observed in patients with known CVDs.^[64]^ Several recent studies have described the effect of statins on serum C-reactive protein levels in patients with CKD. Panichi et al reported that a commonly used dose of simvastatin showed anti-inflammatory effects in patients with CKD.^[65]^ Similarly, Goicoechea et al demonstrated that atorvastatin treatment improved inflammatory status in patients besides showing beneficial effects on the lipid profile.^[66]^ Previous statin trials have shown that reduction in LDL-C results in a proportional decrease in the risk.^[46,59]^ Thus, increasing the statin dose is an effective strategy to increase the benefit to high-risk patients, although it is not always desirable because of drug toxicity concerns, especially when the dose is already high or CKD has progressed. The response to statin therapy in dialysis patients is different from that in the normal population or patients with pre-dialysis CKD. Recent large-scale SHARP, 4D, and AURORA studies have shown that statin therapy had no benefit in dialysis patients in terms of improving primary outcomes (non-fatal myocardial infarction, non-fatal hemorrhagic stroke, non-fatal non-hemorrhagic stroke, and coronary revascularization).^[34,67,68]^ Therefore, the KDOQI guidelines recommended that statin therapy should not be initiated in adult dialysis patients, although it does not need to be stopped if already initiated.^[7]^

This study has some limitations. First, data for certain medications, such as pitavastatin, which have been used recently and for which not many RCTs were available, were not included. Owing to the lack of RCTs for these drugs, information is lacking on the effectiveness of the drugs used for patients with CKD. Second, the effects of drugs on the progression of CKD need to be analyzed. There is a paucity of information on the effects of statins and lipid profiles on the progress of CKD, and further research is needed in this respect. In addition, there is a possibility of a difference between actual conditions and those assessed using estimation formulae without directly examining CKD progression in a patient. Third, there is a lack of information regarding other medications that a patient might be taking simultaneously with statin therapy. Other drugs may affect outcomes if they affect the serum concentration or action of statins. There is currently insufficient evidence that comorbidity of the genetic profile of a patient or underlying disease can cause CVD more frequently.

In conclusion, administration of statins to reduce patient mortality and cardiac events in patients with CKD is an effective and proven therapy. Among the statins currently prescribed, atorvastatin 80 mg, fluvastatin 40 mg, lovastatin 20 mg, pravastatin 40 mg, and simvastatin 40 mg effectively reduced the risk of cardiac events compared with the corresponding placebos. In our network meta-analysis, pravastatin 40 mg showed the highest rank probability for all-cause mortality, whereas for cardiac events, lovastatin 20 mg, fluvastatin 40 mg, and pravastatin 40 mg ranked first, second, and third, respectively. Although there are a variety of statins available for the treatment of patients with CKD, the availability and usage of these agents are often country- or hospital-dependent. Given these circumstances, primary care physicians tend to select medicines based on their previous experience with medication or advice from a senior physician. The findings of this study may prove beneficial for selection of statins to lower the risk of CVD in patients with CKD. Although we cannot provide guidelines for selecting statins that will be the most effective, we anticipate that the data in this study will assist physicians in making informed decisions when selecting statins.

## Author contributions

Conceptualization: HSD, LJH, YJK, and SJH; methodology & data acquisition: HSD, LJH, LSW, YJK, KK, and SJH; data analysis and interpretation: HSD, LJH, YJK, KK, and SJH; statistical analysis: HSD, LJH, and SJH; writing – original draft preparation: HSD, LJH, and SJH; writing – review and editing: HSD, LJH, and SJH; funding acquisition: HSD and SJH.
